# Microbial Epidemiology and Antimicrobial Resistance of Bloodstream Infections in Hospitalized Patients During 2019–2023 in Xiangyang, China

**DOI:** 10.1155/cjid/9969709

**Published:** 2025-11-30

**Authors:** Weiliang Cao, Liang Shen, Jianzhong Zhao, Yan Wang, Fengyu Liu, Jingyuan Huo, Juan Zhou, Li Shi, Chunhua Wang

**Affiliations:** ^1^Department of Clinical Laboratory, Xiangyang No. 1 People's Hospital, Hubei University of Medicine, Xiangyang 441000, Hubei Province, China; ^2^Department of Central Laboratory, Xiangyang Central Hospital, Affiliated Hospital of Hubei University of Arts and Science, Xiangyang 441021, Hubei Province, China; ^3^Experimental Research Center, Capital Center for Children's Health, Capital Medical University, Capital Institute of Pediatrics, Beijing 100020, China

**Keywords:** antimicrobial resistance, bloodstream infection, microbial epidemiology

## Abstract

Bloodstream infections (BSIs) remain a global health threat with high mortality, compounded by shifting pathogen distributions and rising antimicrobial resistance. It is vitally important to clarify the epidemiology and antibiotic resistance transition of BSI pathogens. This retrospective study analyzed clinical data, pathogen profiles, and antimicrobial susceptibility of BSI inpatients from 2019 to 2023 in Xiangyang, China. Among 2312 isolates, Gram-negative bacteria (52.4%) predominated over Gram-positive bacteria (45.5%), with fungi comprising 2.1%. The prevalence of Gram-negative bacteria showed an increasing trend, while that of Gram-positive bacteria declined. Predominant pathogens included *Staphylococcus hominis*, *Staphylococcus epidermidis*, *Staphylococcus aureus*, *Escherichia coli*, *Klebsiella pneumoniae*, and *Pseudomonas aeruginosa*. Notably, 37 cases of polymicrobial BSIs were identified. *S. aureus*, *E. coli*, and *K. pneumoniae* exhibited high resistance to most common antibiotics. Although resistance rates of methicillin-resistant *S. aureus* and carbapenem-resistant *K. pneumoniae* have decreased, they remain substantial. Conversely, carbapenem-resistant *E. coli* prevalence increased annually. The microbes causing BSI are diverse, with varying epidemic characteristics and antibiotic resistance trends. Continuous monitoring of BSI pathogen epidemiology and resistance trends is essential to guide empirical therapy and control resistant strain transmission.

## 1. Introduction

Bloodstream infection (BSI) is a severe systemic infectious disease caused by the invasion of pathogenic microorganisms into the blood circulation system, with high morbidity and mortality. Patients are usually characterized by fever, chills, hypotension, and shock [1, 2]. BSI not only prolongs hospital stays to increase economic burdens but also could lead to multiple organ dysfunction and even death [3].

Many factors contribute to the occurrence of BSI, including immunocompromised states, underlying chronic diseases, invasive procedures, and antibiotic misuse. In recent years, the global aging population has resulted in an increase in the number of elderly hospitalized patients with weakened immune systems or chronic comorbidities, thereby elevating their risk of developing BSI [4]. Additionally, the widespread implementation of invasive surgical procedures has led to a rising incidence of BSI among surgical patients worldwide. The irrational use of antibiotics has exacerbated the issue of antimicrobial resistance, with the prevalence of multidrug-resistant pathogens among BSI patients becoming increasingly concerning. Furthermore, the BSI patient's condition is often complex and rapidly evolving, which has significantly increased the risk of death in BSI patients infected with multidrug-resistant pathogens [5]. Therefore, monitoring the epidemiology of pathogens responsible for BSI and their antimicrobial resistance is crucial for accurate diagnosis, timely treatment, and improving prognosis.

The distribution and drug resistance of BSI pathogens are usually different in times, populations, regions, and even hospitals. BSI patients are predominantly caused by Gram-positive bacteria in East China, while they are more commonly caused by Gram-negative bacteria in Central China, with changes in different age groups and seasons [6–8]. Although Gram-negative bacteria have been reported to be the main pathogen of BSI abroad, the strain composition and dominant epidemic strain of Gram-negative bacteria differ from those of China [9, 10]. So the complexity of the BSI pathogen spectrum and the dynamic characteristics of its epidemiology pose significant challenges for clinical treatment. Moreover, the misuse of antimicrobial agents exacerbates the occurrence of drug resistance of BSI pathogens and could induce multiple resistance mechanisms for pathogens adapting to their environment, complicating empirical antimicrobial therapy [11–14]. Furthermore, the emergence and prevalence of multidrug-resistant pathogens further escalate the complexity of BSI patients' treatment. *Staphylococcus aureus* (Sau), *Klebsiella pneumoniae* (Kpn), *Acinetobacter baumannii* (Aba), *Pseudomonas aeruginosa* (Pae), and other *Enterobacter* members easily develop multidrug resistance in BSI hospitalized patients, enabling them to withstand the bactericidal effects of various antimicrobial agents [15, 16].

Continuous and localized epidemiological surveillance is crucial, as pathogen distribution and resistance patterns are dynamic and vary significantly by region. Data on the recent epidemiological trends of BSIs, particularly covering the period of the coronavirus disease 2019 (COVID-19) pandemic in Central China, are limited. Therefore, this study aimed to investigate the etiological spectrum, epidemiological characteristics, and antimicrobial resistance trends of BSIs among hospitalized patients in Xiangyang, Central China, from 2019 to 2023. The findings will provide updated evidence to inform local empirical antibiotic therapy and infection control strategies.

## 2. Methods

### 2.1. Data Collection

The data of clinical information, pathogenic identification, and antimicrobial susceptibility testing of hospitalized patients with BSI from two tertiary hospitals in Xiangyang City of Hubei Province were collected from 2019 to 2023. The BSI patients enrolled here must meet the following criteria: (a) hospitalized patients with BSI meet the diagnostic criteria outlined in the *Diagnostic and Clinical Treatment Guidelines for Bloodstream Infections* [17]. (b) the same patient with identical strains obtained during the same treatment cycle will not be included repeatedly. (c) patient detected with coagulase-negative staphylococci (CoNS) positive only in a single vial of a set or multiple sets of blood cultures was excluded, unless that CoNS isolated from multiple sterile sites or deemed clinically significant by a physician based on specific symptoms and signs (e.g., fever, systemic inflammatory response) was enrolled. Additionally, all blood cultures flagged as contaminated by the laboratory according to standard protocols (e.g., growth of *Micrococcus* spp., *Bacillus* spp., or mixed flora) were excluded.

### 2.2. Microbe Culture

Pathogenic microbial culture was performed according to the *Clinical Microbiology Laboratory Blood Culture Operation Guidelines* (WS/T 503–2017) [18]. In brief, 1–5 mL (child) or 8–10 mL (adult) of aseptically collected venous blood was injected into appropriate blood culture vials, followed by quickly placing them in the BACTEC FX automated blood culture system (BD Biosciences, USA). The positive blood culture vials were retrieved when the instrument indicated positive alarms, and the culture fluids were smeared for microscopy. Meanwhile, the culture fluids were subcultured onto blood agar and chocolate agar plates and incubated at 35°C for 18–24 h in a CO_2_ incubator that maintained an atmosphere of 5% CO_2_, followed by microbe identification and antimicrobial susceptibility testing.

### 2.3. Microbe Identification and Antimicrobial Susceptibility Testing

The microbial strains were identified using Bruker Microflex LT matrix-assisted laser desorption ionization time-of-flight mass spectrometry (MALDI-TOF MS) systems (Bruker Biosciences Corporation, Germany) [19], and in vitro antimicrobial susceptibility testing was performed using the BD Phoenix M50 automated microbiology system (BD Biosciences, USA). The interpretation of susceptibility results was conducted according to the *Performance Standards for Antimicrobial Susceptibility Testing M100* (CLSI M100.2019) [20]. The quality control strains, including *Escherichia coli* (ATCC 25922), *S. aureus* (ATCC 29213), *P. aeruginosa* (ATCC 27853), and *Enterococcus faecalis* (ATCC 29212), were purchased from the National Center for Clinical Laboratories.

### 2.4. Statistical Analysis

Data statistical analysis was performed using Whonet 5.6, GraphPad Prism 8.0.2, and R (Version 4.2.1) software. Enumeration data were shown as percentages (%) and comparisons between two groups were conducted using the *χ*^2^ test. To identify the independent risk factors distinguishing Gram-positive bacterial from Gram-negative bacterial BSIs, three separate multivariate logistic regression models were established. These models were used to analyze the effects of age, gender, and department, respectively, with all models adjusted for year as a confounding variable. The dependent variable was dichotomized, where Gram-positive bacterial infection was coded as 1 and Gram-negative bacterial infection (serving as the reference group) as 0. The reference categories were specified as follows: 18–60 years for age, female for gender, and internal medicine for department. Results are reported as adjusted odds ratios (OR) with their corresponding 95% confidence intervals (95% CI). A two-tailed *p-*value of less than 0.05 was deemed statistically significant.

## 3. Results

### 3.1. Pathogenic Microbial Distribution of BSI Patients

A total of 2312 pathogenic strains were isolated from 2191 BSI patients during 2019–2023, consisting of 1053 Gram-positive bacteria (45.5%), 1211 Gram-negative bacteria (52.4%), and 48 fungi (2.1%). The detection rate of Gram-positive bacteria from male patients was significantly higher than that from female patients (*p* = 0.032). The highest detection rate of Gram-positive bacteria was found in patients under 18 years old (52.7%), followed by those over 60 years old (30.4%) and those aged 18–60 years old (16.9%). Conversely, the detection rate of Gram-negative bacteria from male patients was lower than that from female patients. The highest detection rate of Gram-negative bacteria was found in patients over 60 years old (62.4%), followed by those aged 18–60 years old (32.9%) and under 18 years old (4.7%). There were significant differences in the detection rate of both Gram-positive and Gram-negative bacteria among the three age groups (*p* = 0.009 and *p* < 0.0001, respectively). Gram-positive and Gram-negative bacteria detected from BSI patients were distributed across 26 and 30 clinical departments, respectively (Table S1). Among them, the top 5 departments with a detection rate of Gram-positive bacteria were pediatrics, internal medicine, intensive care unit, neonatology, and surgery, and the top 5 departments with a detection rate of Gram-negative bacteria were internal medicine, intensive care unit, surgery, obstetrics and gynecology, and pediatrics (Table 1).

### 3.2. Composition and Epidemic Characteristic of Gram-Positive Bacteria Spectrum

**Figure 1:**
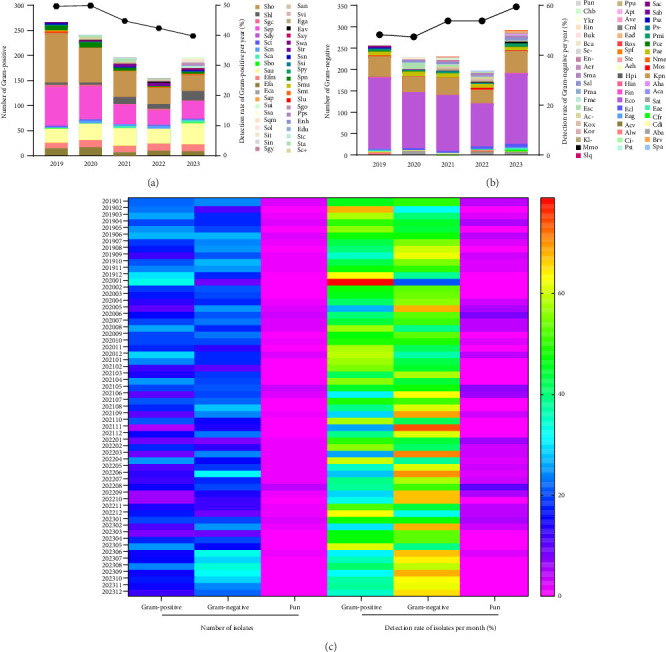
The species abundance distribution and epidemic characteristic of pathogenic microbes caused BSI during 2019–2023. (a) The species composition and detection rate of Gram-positive bacteria in BSI. (b) The species composition and detection rate of Gram-positive bacteria in BSI. (c) The epidemic trend of pathogenic microbes in BSI during 2019–2023. G+, Gram-positive bacteria; G-, Gram-negative bacteria; Fun, fungus.

A total of 42 strains of Gram-positive bacteria were isolated from the blood of BSI patients, including Sau, *Staphylococcus haemolyticus* (Shl), *Staphylococcus epidermidis* (Sep), *Staphylococcus saprophyticus* (Sap), *Staphylococcus hominis* (Sho), *Streptococcus pyogenes* (Spy), *Streptococcus agalactiae* (Sgc), *Enterococcus faecium* (Efm), *Enterococcus faecalis* (Efa), *Streptococcus suis* (Sui), *Streptococcus gallolyticus* (Sgy), and *Streptococcus pneumoniae* (Spn) (Table S2). The top 6 isolates were Sho (26.9%), Sep (23.0%), Sau (16.0%), Efm (6.0%), Efa (5.1%), and Shl (5.0%). Notably, the isolation rates of CoNS, such as Sho and Sep, might be higher than that in actuality, as several colonizing or contaminating strains might not be thoroughly excluded. From 2019 to 2023, the detection rate of Gram-positive bacteria decreased annually, but the abundance of Gram-positive bacterial composition increased. Especially, the abundance of Gram-positive bacterial composition was up to 32 species in 2023 (Figure 1(a)). Additionally, BSIs caused by Gram-positive bacteria were prevalent throughout the year, with a peak in autumn and winter (Figure 1(c)).

### 3.3. Composition and Epidemic Characteristic of Gram-Negative Bacteria Spectrum

A total of 58 strains of Gram-negative bacteria were isolated from the blood of BSI patients (Table S3), including *Escherichia coli* (Eco), *Enterobacter cloacae* (Ecl), Kpn, Pae, Aba, *Acinetobacter lwoffii* (Alw), *Klebsiella oxytoca* (Kox), *Stenotrophomonas maltophilia* (Pma), *Citrobacter freundii* (Cfr), *Haemophilus influenzae* (Hin), *Neisseria meningitidis* (Nme), *Proteus mirabilis* (Pmi), and *Moraxella* (Branh) *Catarrhalis* (Bca). The top 6 isolates were Eco (57.1%), Kpn (17.3%), Pae (3.9%), Ecl (2.1%), Aba (2.1%), and Pmi (1.5%). From 2019 to 2023, the detection rate of Gram-negative bacteria increased overall, as well as their abundance. Especially, the abundance of Gram-negative bacterial composition was up to 32 species in 2023 (Figure 1(b)). Additionally, BSIs caused by Gram-negative bacteria were prevalent throughout the year, with a peak in summer, autumn, and winter (Figure 1(c)).

### 3.4. Concurrent Infections of Multiple Pathogenic Bacteria-Caused BSI

A total of 37 cases were found to be co-infected with two or more pathogenic microbes in 2191 patients with BSI. The incidence of dual infection with two pathogenic microbes was as high as 97.3% (36/37). The combinations of dual infection was diverse, including Gram-positive bacteria paired with Gram-positive bacteria (Sho co-infected with Shl, Efa co-infected with Sau, Sxy co-infected with Efa), Gram-negative bacteria paired with Gram-negative bacteria (Eco co-infected with Kpn, Eco co-infected with Pma, Eco co-infected with Aba), Gram-positive bacteria paired with Gram-negative bacteria (Sau co-infected with Kpn, Efa co-infected with Eco, Efm co-infected with Eco, Efm co-infected with Pae, Efm co-infected with Kpn, Efa co-infected with Pmi), Gram-negative bacteria paired with fungi (Kpn co-infected with Ctr), Gram-positive bacteria paired with fungi (Efa co-infected with Cal), and fungi paired with fungi (Cal co-infected with Ctr). Additionally, only one case was found to be triple infected with Sau, Efa, and Aba.

### 3.5. Antimicrobial Resistance Transition of Major Gram-Positive Bacteria in BSI

**Figure 2:**
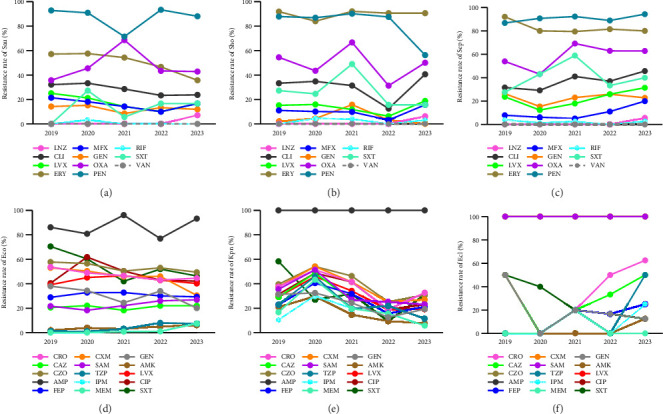
The drug resistance transition of major Gram-positive and Gram-negative bacteria spectrum in BSI during 2019–2023. (a) The resistance change of Sau to commonly used antibiotics. (b) The resistance change of Sho to commonly used antibiotics. (c) The resistance change of Sep to commonly used antibiotics. (d) The resistance change of Eco to commonly used antibiotics. (e) The resistance change of Kpn to commonly used antibiotics. (f) The resistance change of Ecl to commonly used antibiotics.

Varying degrees of resistance to commonly used antibiotics (vancomycin excluded) were shown in major Gram-positive bacteria of BSI after the analysis of antimicrobial susceptibility testing results (Table 2). Sau, Sho, Sep, and Shl had higher 5-year resistance rates to penicillin, erythromycin, and oxacillin, among which the resistance rates to penicillin exceeded 84%. Apart from Shl, the other three *Staphylococcus* species maintained low resistance rates to linezolid and rifampin (< 3%). The resistance rates of Sau and Sho to more than half of the commonly used antibiotics showed a decreasing trend annually (Figures 2(a), 2(b)), while that of Sep exhibited the opposite trend (Figure 2(c)). The resistance rates of Efm to penicillin and ampicillin both exceeded 80%, as well as that of Efa to ampicillin and GEH, which both exceeded 40%. The resistances to linezolid and vancomycin were not found in enterococcus.

### 3.6. Antimicrobial Resistance Transition of Major Gram-Negative Bacteria in BSI

Similar to Gram-positive bacteria, varying degrees of resistance to commonly used antibiotics were also shown in major Gram-negative bacteria of BSI after the analysis of antimicrobial susceptibility testing results (Table 3). Enterobacteriaceae (Eco, Kpn, Ecl, and Pmi) exhibited high resistance rates to ampicillin, first- to fourth-generation cephalosporins, fluoroquinolones (levofloxacin and ciprofloxacin), and trimethoprim-sulfamethoxazole. However, their resistance rates to carbapenems (imipenem and meropenem) were relatively low, and resistance to imipenem and meropenem was not found in Pmi. Furthermore, the resistance rates of Eco and Kpn to commonly used antibiotics (ampicillin excluded) showed a decreasing trend annually (Figures 2(d), 2(e)), while that of Ecl exhibited the opposite trend (Figure 2(f)). Pae showed high sensitivity to commonly used antibiotics (fluoroquinolones excluded), while Aba displayed high levels of resistance to commonly used antibiotics, with resistance rates exceeding 68%.

### 3.7. Epidemic Characteristics of Multidrug-Resistant Bacteria in BSI

A total of 158 strains of multidrug-resistant bacteria were identified from 2019 to 2023, accounting for 6.8% and consisting of multidrug-resistant Sau and multidrug-resistant Gram-negative bacteria. The ranking of resistance rates from high to low was as follows: carbapenem-resistant Aba (CRAB, 18/25, 72%), methicillin-resistant Sau (MRSA, 80/168, 47.6%), carbapenem-resistant Kpn (CRKP, 40/210, 19%), carbapenem-resistant Ecl (CREC, 3/26, 11.5%), carbapenem-resistant Pae (CRPA, 3/47, 6.3%), and carbapenem-resistant Eco (14/692, 2%). Notably, the rate of carbapenem-resistant Eco had shown an increasing trend annually.

### 3.8. Risk Factors for Gram-Positive Bacterial vs. Gram-Negative Bacterial BSIs

Distinct risk factors for Gram-positive bacterial versus Gram-negative bacterial BSIs were identified by multivariate logistic regression analysis (Table 4). The data revealed that young patients (< 18 years) demonstrated substantially increased susceptibility to Gram-positive bacterial BSIs compared to the 18–60-year-old reference group (OR = 22.97, 95% CI: 16.63–32.23, *p* < 0.001). However, patients over 60 years showed no significant difference in Gram-positive bacterial BSI risk compared to the reference group (OR = 0.94, 95% CI: 0.75–1.17, *p* = 0.575). Furthermore, male patients exhibited significantly higher odds of Gram-positive bacterial BSIs compared to females (OR = 1.71, 95% CI: 1.45–2.03, *p* < 0.001). Substantial departmental variations were also observed, with pediatric departments showing extremely elevated odds of Gram-positive bacterial infections (OR = 33.81, 95% CI: 22.70–52.14, *p* < 0.001), followed by neonatology departments (OR = 14.30, 95% CI: 8.31–26.28, *p* < 0.001). The ICU department demonstrated moderately increased odds (OR = 1.45, 95% CI: 1.10–1.91, *p* = 0.008), while the surgery and obstetrics-gynecology departments showed reduced Gram-positive bacterial infection odds.

## 4. Discussion

Bacterial resistance is an increasingly serious public health issue, with great concern surrounding BSI caused by resistant bacteria. In this study, a retrospective analysis was conducted on the data of clinical information, pathogenic identification, and antimicrobial susceptibility testing in hospitalized patients with BSI from 2019 to 2023. The isolation rate of Gram-positive bacteria with BSI was 45.5% in Xiangyang, which was slightly different from that in Tianjin (39.8%) and Wuhan (48.1%) [21, 22], indicating regional differences in the distribution of Gram-positive bacteria causing BSI. Notably, Gram-positive bacteria were mainly isolated from pediatric patients with BSI, which may be related to the unsound immune system of children, low immunity, and easy occurrence of upper respiratory tract infection [23]. Moreover, the detection rate of Gram-positive bacteria in males was significantly higher than that in females (*p* < 0.05), suggesting that gender may be an important risk factor for the occurrence of BSI caused by Gram-positive bacteria. Further research needs to validate the point with a larger sample size of BSI caused by Gram-positive bacteria.

In the spectrum of Gram-positive bacteria, *Staphylococcus* occupied 70.8%. Of them, Sho (a CoNS) was most commonly prevalent in BSI in the present study. CoNS are major colonized bacteria on human skin, in the oral cavity, and in the intestines. They can invade abnormal colonization sites to cause BSI when the immune function of the host is compromised or during invasive manipulations [24]. Moreover, they also can lead to false-positive blood cultures if blood culture specimens are not properly or thoroughly disinfected prior to collection, as CoNS on skin can easily contaminate the culture bottles. Therefore, the diagnosis of BSI caused by CoNS must consider the clinical manifestations of patients. The declining trend in Gram-positive bacterial BSI detection (2019–2023) reflects an epidemiological shift; the result is consistent with the findings of Orsi et al. [25], likely influenced by changes in antibiotic prescribing, infection control, or microbial ecology during the COVID-19 pandemic. However, the abundance of Gram-positive bacteria causing BSI has been increasing, indicating the diversity and complexity of pathogens responsible for BSI. Meanwhile, certain bacteria may have acquired the pathogenic ability to cause BSI following the COVID-19 pandemic. Additionally, Efm is one of the significant pathogens causing BSI; it shows high resistance to penicillin and ampicillin (> 80%), consistent with previous reports [26]. Multivariate logistic regression identified independent risk factors for Gram-positive bacterial versus Gram-negative bacterial BSIs. Young populations (OR = 22.97 for those under 18 years) showed higher Gram-positive bacteria infected odds than the 18–60-year-old reference group. The result is consistent with the research of Shao H et al. [27], suggesting age-specific immunological factors or exposure patterns influence BSI etiology. Departmental clustering was prominent, particularly in pediatrics (OR = 33.81) and neonatology (OR = 14.3), underscoring the need for department-specific infection control strategies and empirical antibiotic selection [27]. A moderate but significant gender disparity (71% higher odds in males) warrants further investigation into biological or behavioral contributors. These underscore the necessity for ongoing surveillance and potential revision of empirical treatment guidelines.

Gram-negative bacteria are usually the main pathogens causing BSI [20–22]. In this study, the detection rate of Gram-negative bacteria was up to 52.4%, distributed across internal medicine, intensive care unit, surgery, obstetrics and gynecology, and pediatric departments. The detection rates of Gram-negative bacteria in the former two departments were significantly higher than that in others (*p* < 0.01). Most patients in these two departments were elderly, and the incidence of BSI in patients over 60 years old was significantly higher than that in patients under 18 years old (*p* < 0.01), suggesting that advanced age may be a risk factor for Gram-negative bacteria-caused BSI. The incidence of BSI continues to rise with the increasing elderly population, as has been indicated in a previous study [28]. The fluctuation in pathogen distribution appears to be associated with the COVID-19 pandemic. During the pandemic period (2019–2022), the detection rates of Gram-negative in BSI patients showed a downward trend, which may be attributed to enhanced infection control measures, widespread mask-wearing, and reduced hospital visits for non-urgent conditions. The sharp rebound in 2023, following the relaxation of strict public health measures, suggests a resurgence of community-acquired and healthcare-associated BSIs. This pattern underscores the profound impact of public health interventions on the epidemiology of infectious diseases. Furthermore, the higher temperature or humidity might provide favorable conditions for the growth and reproduction of Gram-negative bacteria, resulting in the Gram-negative bacteria-caused BSI having a prevalent peak in summer and autumn. Additionally, Gram-negative bacteria are easily resistant to a variety of antibiotics. Imipenem, meropenem, amikacin, and piperacillin–tazobactam could be used as candidate therapy drugs for Eco-caused BSI, as the resistance rates to these drugs of Eco are maintained at a low level. However, Kpn and Aba were found to be intermediate or completely resistant to most common antibiotics, which might be a result of advanced age, multiple hospitalizations (especially in ICU), long-term use of antibiotics, or combined underlying diseases [29]. Therefore, the management of BSI patients caused by Kpn and Aba should be strengthened to prevent hospital-acquired infections.

In this study, many co-infection events were found with diverse patterns. The interaction between different pathogenic bacteria may affect the severity and prognosis of infection [30]. In particular, the Gram-negative bacteria paired with the Gram-negative bacteria pattern, such as Eco and Kpn co-infection, may indicate the inappropriate use of antibiotics or aggravation of drug resistance, making the clinical treatment of co-infections complicated [31, 32]. The Gram-positive and Gram-negative bacteria co-infection, such as Sau and Kpn, suggests that complex mechanisms of infection may exist in severe patients, making clinical management a huge challenge. In addition, fungi could co-infect with Gram-negative bacteria, Gram-positive bacteria, and other fungi, which usually leads to serious consequences if not treated in time [33]. Clinicians should pay more attention to fungal infections and strengthen monitoring of high-risk BSI patients.

The epidemic rates of MRSA, CRAB, CRPA, carbapenemase-resistant Enterobacteriaceae (CRE), and vancomycin-resistant *Enterococcus* (VRE) were monitored according to the national bacterial resistance monitoring technical program of China. The results revealed that CRAB was the highest (72%) during the COVID-19 pandemic, indicating that the resistance of this strain to commonly used antibiotics was significantly enhanced, which may be attributed to its efflux pump-mediated resistance mechanisms [34]. Meanwhile, the rate of CRAB in this study was higher than that reported in a recent study from East China (61.20%–66.60%) [35] and some European countries (41.6%) [36]. Additionally, MRSA, with a detection rate of 47.6%, should warrant attention due to its frequent occurrence and severe impact in clinical BSIs, often leading to poor treatment outcomes [37]. This MRSA infection rate also exceeds that reported in many European countries (approximately 25%) [38] but aligns with data from southern China [39]. Additionally, the prevalence of CRKP and CREC should not be ignored, especially since CRKP has become one of the important factors in hospital-acquired infections worldwide [40]. The detection rate of CRKP (19%) was comparable to that reported in Southwest China [41]. However, the detection rate of CRE (11.5%) was lower than that reported in a recent study from Southwest China (approximately 40%) [41], but comparable to that reported in East China [26]. This highlights the regional variability in multidrug-resistant organism (MDRO) prevalence and underscores the need for local antimicrobial stewardship programs.

This study has several limitations. The high proportion of CoNS (such as Sho and Sep) among Gram-positive bacteria should warrant caution. Although we implemented strict exclusion criteria to rule out contaminants, it was possible that several colonizing or contaminating strains were included, as the clinical significance of a single vial of a set or multiple sets of blood cultures for CoNS-positive could be difficult to ascertain retrospectively. The prevalence and clinical impact of CoNS-reported BSIs might be overestimated. Future prospective studies with predefined clinical criteria for true CoNS infection are needed to validate the findings. In addition, this study was conducted in one tertiary hospital in a single city in Central China. The findings regarding pathogen distribution and resistance patterns may not be directly generalizable to other geographical regions of China. Multicenter studies involving diverse regions are warranted to obtain a more comprehensive national perspective.

## 5. Conclusions

In summary, there is a wide variety of pathogens causing BSI, with complex epidemiological characteristics and diverse resistance phenotypes. Strengthening the monitoring of the pathogen spectrum and the resistance of commonly used antimicrobial agents in BSI can provide proper guidance for clinically rational antimicrobial therapy.

## Figures and Tables

**Table 1:** The pathogenic microbial distribution of BSI during 2019–2023.

Variables	Number of pathogenic microbe strains						*P*
	2019	2020	2021	2022	2023	Total, N (%)	
Gram-positive bacteria							
** **Age, years							
** **< 18	190	142	108	67	48	555 (52.7%)	0.009
** **18–60	27	27	46	29	49	178 (16.9%)	
** **> 60	49	72	41	59	99	320 (30.4%)	
Gender							
** **Female	107	100	64	60	87	418 (39.7%)	0.032
** **Male	159	141	131	95	109	635 (60.3%)	
Department							
** **PED	157	102	80	52	41	432 (41.0%)	0.003
** **MED	46	55	36	43	12	192 (18.2%)	
** **ICU	31	30	20	30	42	153 (14.5%)	
** **NEO	18	38	25	13	2	96 (9.1%)	
** **SUR	12	9	23	14	4	62 (5.9%)	
Gram-negative bacteria							
** **Age, years							
** **< 18	16	11	9	9	12	57 (4.7%)	< 0.0001
** **18–60	84	78	80	63	93	398 (32.9%)	
** **> 60	158	140	145	125	188	756 (62.4%)	
Gender							
** **Female	137	125	116	100	164	642 (53.0%)	0.421
** **Male	121	104	118	97	129	569 (47.0%)	
** **Department							
** **MED	126	93	112	86	17	434 (35.8%)	0.0009
** **ICU	52	49	33	46	52	232 (19.2%)	
** **SUR	43	51	64	38	9	205 (16.9%)	
** **OBG	9	8	5	9	11	42 (3.5%)	
** **PED	8	6	1	6	8	29 (2.4%)	

Abbreviations: PED, pediatric department; MED, internal medicine department; ICU, intensive care unit; NEO, neonatology department; SUR, surgery department; OBG, obstetrics and gynecology department.

**Table 2:** The spectrum and change of drug resistance to major Gram-positive bacteria in BSI.

Antibiotics	Resistance rate (%)					
	Sau (*n* = 168)	Sho (*n* = 283)	Sep (*n* = 242)	Shl (*n* = 53)	Efa (*n* = 54)	Efm (*n* = 63)
PEN	86.9	84.5	90.1	96.2	37.0	93.7
ERY	49.4	89.8	83.9	83.0	NA	NA
OXA	47.6	50.9	55.8	84.9	NA	NA
LNZ	1.8	0.7	0.8	3.8	0.0	0.0
CLI	27.9	31.8	35.1	52.8	NA	NA
LVX	17.3	14.1	21.1	71.7	NA	NA
MFX	16.1	10.2	9.1	52.8	NA	NA
RIF	0.6	2.1	2.5	26.4	NA	NA
SXT	13.7	27.9	39.3	20.8	NA	NA
GEN	12.5%	4.9	22.3	43.4	NA	NA
VAN	0.0	0.0	0.0	0.0	0.0	0.0
AMP	NA	NA	NA	NA	40.7	80.9
GEH	NA	NA	NA	NA	50.0	38.1
STH	NA	NA	NA	NA	29.6	39.7

**Table 3:** The spectrum and change of drug resistance to major Gram-negative bacteria in BSI.

Antibiotics	Resistance rate (%)					
	Eco (*n* = 692)	Kpn (*n* = 210)	Ecl (*n* = 26)	Pmi (*n* = 18)	Pae (*n* = 47)	Aba (*n* = 25)
AMP	87.4	100.0	100.0	50.0	NA	NA
CAZ	21.0	30.0	30.8	5.6	0.0	68.0
FEP	30.6	26.2	15.4	16.7	0.0	68.0
TZP	3.9	23.3	19.2	0.0	0.0	72.0
SAM	23.0	31.9	100.0	61.1	NA	72.0
CZO	53.5	39.0	100.0	61.1	NA	NA
CXM	44.8	36.7	100.0	38.9	NA	NA
CRO	47.7	36.7	38.5	38.9	NA	NA
IPM	2.0	15.2	11.5	0.0	4.3	72.0
MEM	2.0	19.0	3.8	0.0	4.3	72.0
ATM	NA	NA	NA	NA	2.1	NA
TOB	NA	NA	NA	NA	4.3	NA
GEN	29.9	24.3	15.4	38.9	NA	84.0
AMK	3.9	16.2	3.8	11.1	NA	68.0
LVX	42.5	29.0	19.2	72.2	10.6	76.0
CIP	47.1	32.4	19.2	66.7	10.6	88.0
SXT	54.8	33.3	23.1	77.8	NA	76.0

**Table 4:** The multivariate logistic regression analysis of the risk factors for Gram-positive bacterial versus Gram-negative bacterial BSIs during 2019–2023.

Variable	Adjusted OR	95% CI	*p*-value
Age			
** **< 18 years	22.97	(16.63–32.23)	< 0.001
** **18–60 years (reference)	1.00	—	
** **> 60 years	0.94	(0.75–1.17)	0.575
Gender			
** **Female (reference)	1.00	—	
** **Male	1.71	(1.45–2.03)	< 0.001
Department			
** **PED	33.81	(22.70–52.14)	< 0.001
** **ICU	1.45	(1.10–1.91)	0.008
** **MED (reference)	1.00	—	
** **NEO	14.3	(8.31–26.28)	< 0.001
** **SUR	0.68	(0.49–0.95)	0.024
** **OBG	0.52	(0.24–1.03)	0.075

## Data Availability

All data are contained within the article.

## Supporting Information

Table S1: Department distribution of pathogenic microbes isolated from BSI hospitalized patients during 2019–2023.

Table S2: Species and numbers of Gram-positive bacteria spectrum isolated from BSI patients.

Table S3: Species and numbers of Gram-negative bacteria spectrum isolated from BSI patients.
