# Supplementary material for: Microbial Epidemiology and Antimicrobial Resistance of Bloodstream Infections in Hospitalized Patients During 2019–2023 in Xiangyang, China
Source: Can J Infect Dis Med Microbiol. 2025 Nov 30;2025:9969709. doi: 10.1155/cjid/9969709 (PMC12682447; doi:10.1155/cjid/9969709)
Supplement: Supporting Information — Additional supporting information can be found online in the Supporting Information section. [file 9969709.f1.pdf]

Microbial Epidemiology and Antimicrobial Resistance of Bloodstream Infections in Hospitalized Patients During 2019-2023

Weiliang Cao[1†], Liang Shen[2†], Jianzhong Zhao[1†], Yan Wang[1], Fengyu Liu[1], Jingyuan Huo[1], Juan Zhou[3], Li Shi[1]*, Chunhua Wang[1,2]*

[1] Department of Clinical Laboratory, Xiangyang No. 1 People's Hospital, Hubei University of Medicine, Xiangyang, 441000, Hubei Province, China

[2] Department of Central Laboratory, Xiangyang Central Hospital, Affiliated Hospital of Hubei University of Arts and Science, Xiangyang, 441021, Hubei Province, China

[3] Experimental Research Center, Capital Center for Children's Health, Capital Medical University, Capital Institute of Pediatrics, Beijing 100020, China

Table S1 Department distribution of pathogenic microbes isolated from BSI hospitalized patients during 2019-2023.

Department	Gram-positive bacteria					Gram-negative bacteria				
	**2019** (n=266)	**2020** (n=241)	**2021** (n=195)	**2022** (n=155)	**2023** (n=196)	**2019** (n=258)	**2020** (n=229)	**2021** (n=234)	**2022** (n=197)	**2023** (n=293)
ped	157	102	80	52	41	8	6	1	6	8
icu	31	30	20	30	42	52	49	33	46	52
med	46	55	36	43	12	126	93	112	86	17
neo	18	38	25	13	2	1	5	6	3	0
sur	12	9	23	14	4	43	51	64	38	9
obg	0	2	5	1	2	9	8	5	9	11
oth	0	2	5	2	2	14	15	12	8	0
eme	2	3	1	0	0	3	1	1	0	0
unk	0	0	0	0	0	2	1	0	1	0
res	0	0	0	0	14	0	0	0	0	23
nep	0	0	0	0	13	0	0	0	0	18
onc	0	0	0	0	13	0	0	0	0	23
gas	0	0	0	0	11	0	0	0	0	9
neu	0	0	0	0	8	0	0	0	0	19
reh	0	0	0	0	7	0	0	0	0	5
ort	0	0	0	0	4	0	0	0	0	4
end	0	0	0	0	3	0	0	0	0	4
hao	0	0	0	0	3	0	0	0	0	2

rme	0	0	0	0	3	0	0	0	0	12
car	0	0	0	0	2	0	0	0	0	0
hem	0	0	0	0	2	0	0	0	0	11
inf	0	0	0	0	2	0	0	0	0	19
urs	0	0	0	0	2	0	0	0	0	24
ctsu	0	0	0	0	1	0	0	0	0	0
dis	0	0	0	0	1	0	0	0	0	0
gyn	0	0	0	0	1	0	0	0	0	0
out	0	0	0	0	1	0	0	0	0	0
hep	0	0	0	0	0	0	0	0	0	7
ger	0	0	0	0	0	0	0	0	0	4
bps	0	0	0	0	0	0	0	0	0	3
car	0	0	0	0	0	0	0	0	0	3
dis	0	0	0	0	0	0	0	0	0	2
nes	0	0	0	0	0	0	0	0	0	2
der	0	0	0	0	0	0	0	0	0	1
rhe	0	0	0	0	0	0	0	0	0	1

Table S2 Species and numbers of gram-positive bacteria spectrum isolated from BSI patients.

Species	2019 (n=266)	2020 (n=241)	2021 (n=195)	2022 (n=155)	2023 (n=196)
sho	99	69	51	32	32
sep	76	65	39	27	35
sau	28	33	35	30	42
efm	10	14	12	14	13
efa	14	16	7	9	8
shl	5	5	14	10	19
spn	9	11	6	2	4
scn	1	5	4	6	2
str	1	2	5	6	0
sgc	4	3	1	3	1
sca	1	1	4	0	4
sta	0	3	6	0	1
stc	0	2	2	1	4
smt	3	0	1	2	2
ssn	3	0	2	0	2
sct	2	2	0	1	1
sdy	2	1	0	2	1
san	0	2	1	2	0
sc+	0	3	1	1	0
eca	1	1	0	1	1

swa	1	0	0	0	3
eav	0	1	0	3	0
sap	1	0	1	0	1
sxy	0	1	1	0	1
svi	0	0	1	2	0
sgo	0	0	0	0	3
sit	0	0	0	0	3
sbo	1	0	0	0	1
spy	1	1	0	0	0
ssi	1	0	0	0	1
sin	0	0	0	0	2
sol	0	0	0	0	2
sui	0	0	0	0	2
slu	1	0	0	0	0
smu	1	0	0	0	0
ega	0	0	1	0	0
edu	0	0	0	1	0
enh	0	0	0	0	1
pps	0	0	0	0	1
sgy	0	0	0	0	1
sqm	0	0	0	0	1
ssa	0	0	0	0	1

Table S3 Species and numbers of gram-negative bacteria spectrum isolated from BSI patients.

**Species**	**2019** (n=258)	**2020** (n=229)	**2021** (n=234)	**2022** (n=197)	**2023** (n=293)
eco	166	131	131	100	164
kpn	48	37	41	32	52
pae	9	11	10	9	8
ecl	2	5	5	6	8
aba	1	9	3	6	6
pmi	3	2	4	2	7
esc	0	13	4	0	0
sal	0	4	5	2	6
sat	3	2	3	1	1
sma	0	2	1	5	2
kox	0	0	3	5	1
mmo	0	1	1	1	4
kl-	0	0	4	2	1
alw	3	0	0	3	0
cfr	1	0	0	1	4
eag	2	0	0	2	2
mos	1	0	0	4	1

ac-	0	1	2	3	0
eae	1	0	0	0	4
ps-	2	1	1	1	0
pma	0	1		2	2
en-	0	0	3	0	2
ead	0	0	0	0	5
acv	1	0	0	2	1
cdi	1	1	1	0	1
pce	2	1	0	0	1
brv	2	1	0	0	0
hin	1	0	0	1	1
kor	0	1	1	1	0
ste	0	0	3	0	0
fin	1	0	0	0	1
psz	2	0	0	0	0
sab	2	0	0	0	0
slq	1	0	0	1	0
fme	0	1	1	0	0
aer	0	0	1	1	0
se-	0	0	1	0	1
chb	0	0	2	0	0
hpi	1	0	0	0	0
nme	1	0	0	0	0
sac	1	0	0	0	0
bca	0	1	0	0	0
buk	0	1	0	0	0
ein	0	1	0	0	0
ykr	0	1	1	0	0
pan	0	0	1	0	0
ppu	0	0	1	0	0
aeh	0	0	0	1	0
ci-	0	0	0	1	0
pst	0	0	0	1	0
spa	0	0	0	1	0
aca	0	0	0	0	1
aha	0	0	0	0	1
apt	0	0	0	0	1
ave	0	0	0	0	1
cml	0	0	0	0	1
ros	0	0	0	0	1
spf	0	0	0	0	1

Abbreviations

Sho, staphylococcus hominis; Shl, staphylococcus haemolyticus; Sgc, streptococcus agalactiae; Sep, staphylococcus epidermidis; Sdy, streptococcus dysgalactiae; Sct, streptococcus constellatus; Scn, staphylococcus coagulase negative; Sca, staphylococcus capitis; Sbo, streptococcus bovis; Sau, staphylococcus aureus; Efm, enterococcus faecium; Efa, enterococcus faecalis; Eca, enterococcus casseliflavus; Sap, staphylococcus saprophyticus; Sui, streptococcus suis; Ssa, streptococcus salivarius; Sqm, streptococcus equisimilis; Sol, streptococcus oralis; Sit, staphylococcus intermedius; Sin,streptococcus intermedius; Sgy, streptococcus gallolyticus; San, streptococcus anginosus; Svi, streptococcus viridans; Ega, enterococcus gallinarum; Eav, enterococcus avium; Sxy, staphylococcus xylosus; Swa, staphylococcus warneri; Str, streptococcus sp.; Ssn, streptococcus sanguis; Ssi, staphylococcus simulans; Spy, streptococcus pyogenes; Spn, streptococcus pneumoniae; Smu, streptococcus mutans; Smt, streptococcus mitis; Slu, staphylococcus lugdunensis; Sgo, streptococcus gordonii; Pps, staphylococcus saccharolyticus; Enh, enterococcus hirae; Edu, streptococcus durans; Stc, staphylococcus cohnii; Sta, Staphylococcus sp.; Sc+, staphylococcus coagulase positive. Pan, pantoea sp.; Chb, chryseobacterium sp.; Ykr, yersinia kristensenii; Ein, enterobacter intermedius; Buk, burkholderia sp.; Bca, Moraxella (Branh.) catarrhalis; Se-, serratia sp.; En-, enterobacter sp.; Aer, aeromonas sp.; Sma, serratia marcescens; Sal, salmonella sp.; Pma, stenotrophomonas maltophilia; Fme, chryseobacterium meningosepticum; Esc, escherichia sp.; Ac-, acinetobacter sp.; Kox, klebsiella oxytoca; Kor, raoultella ornitholytica; Kl-, klebsiella sp.; Mmo, morganella morganii; Slq, serratia liquefaciens; Ppu, pseudomonas putida; Apt, acinetobacter pittii; Ave, aeromonas veronii; Cml, citrobacter amalonaticus; Ead, escherichia coli (alkalescens-dispar); Ros; roseomonas sp.; Spf, shewanella putrefaciens; Ste, stenotrophomonas sp.; Aeh, aeromonas hydrophila; Hin,haemophilus influenzae; Fin, flavobacterium indologenes; Eco, escherichia coli; Ecl, enterobacter cloacae; Eag, enterobacter agglomerans; Acv, aeromonas caviae; Alw, acinetobacter lwoffii; Ci-,

citrobacter sp.; Pst, providencia stuartii; Spa, sphingomonas paucimobilis; Sac, salmonella paratyphi c; Sab, salmonella paratyphi b; Psz, pseudomonas stutzeri; Ps-, pseudomonas sp.; Pmi, proteus mirabilis; Pce, burkholderia cepacia; Pae, pseudomonas aeruginosa; Nme, neisseria meningitidis; Mos, moraxella osloensis; Kpn, klebsiella pneumoniae; Aha, acinetobacter haemolyticus; Aca, acinetobacter calcoaceticus; Sat, salmonella typhi; Eae, klebsiella aerogenes; Cfr, citrobacter freundii; Cdi, citrobacter koseri; Aba, acinetobacter baumannii; Brv, Brevundimonas sp.; Hpi, haemophilus parainfluenzae. LNZ, linezolid; CLI, clindamycin; LVX, levofloxacin; ERY, erythromycin; MFX, moxifloxacin; GEN, gentamicin; OXA, oxacillin; PEN, penicillin; RIF, rifampicin; SXT, sulfamethoxazole/trimethoprim; VAN, vancomycin; CRO, ceftriaxone; CAZ, ceftazidime; CZO, cefoperazone; AMP, ampicillin; FEP, cefepime; CXM, cefuroxime; SAM, ampicillin/sulbactam; TZP, piperacillin/tazobactam; IPM, imipenem; MEM, meropenem; AMK, amikacin; CIP, ciprofloxacin. GEH, gentamicin-high; STH, streptomycin-high. ATM, aztreonam; TOB, tobramycin.
